# The Metropolitan Hospital

**Published:** 1904-07-02

**Authors:** 


### THE METROPOLITAN HOSPITAL.

### ACCOMMODATION FOR THE NURSES NEEDED.

"Proper accommodation for the nurses urgently needed" was the keynote of the speeches at the festival dinner of the Metropolitan Hospital which was held at the Whitehall Rooms on June 23rd, under the presidency of Lord Wolverton. First, Lord Wolverton pointed out that on the occasion of a recent visit he made to the hospital it struck him that the accommodation of the nurses was quite inadequate. Though they seemed to him to be perfectly happy he would like to have seen them better housed. Following him Lord Howard de Walden (the chairman) pointed out that amongst the things which, in his opinion, the hospital urgently required were, first and foremost, better quarters for the nurses. Then Mr. Charles J. Thomas, the chairman of the committee of management, took up the tale by stating that the hospital could only provide 111 beds, out of 160, for the use of the patients until they could provide themselves with a separate home for their nurses, because the nurses' quarters had to be placed in a ward which was intended for patients. That this ward was urgently needed in that poor and populous district was a matter of common knowledge. Lord Battersea, one of the treasurers, too, dwelt on this point, and urged that women who had been engaged whilst on duty in administering to the sick should when their work was done, be able to leave the sick-room entirely behind them. He hoped that something would be done in the immediate future to remedy what he considered was a great blot on the Metropolitan Hospital. Finally, Mr. Goodsall, the Senior Surgeon, confided to those present that the Committee of Management had in view an excellent site, close to the hospital, for a Nurses' Home, and he hoped that in a year or two they might be able to acquire that site and get proper quarters for the nursing staff, not only of the hospital as at present constructed, but of the larger hospital which would result when the whole of the hospital site was covered with wards.

Amongst those present at the dinner were, besides those mentioned above, Mr. Leopold de Rothschild, Sir John Heron-Maxwell, Bart., Lieut.-Col. Montefiore, and Mr. H. Allhusen, M.P.

Lord Wolverton, in proposing the toast, "Prosperity to the Hospital," said he did not wish his hearers to go away with the idea that the hospital he was appealing for was in a prosperous and happy condition. On the contrary, it was in a far from prosperous state. It had to supply the wants and needs of a district containing over 500,000 people, and there was not another hospital within two miles of it.

Mr. A. H. E. Allhusen, M.P., proposed the "Committee of Management," who were he said, so to speak, the silent machinery of the hospital. They worked on steadily year in and year out, and the general public—nay the patients themselves—hardly knew that the committee existed. They received no thanks, and no recognition of any kind though their duties were arduous and many. It was, therefore, the more incumbent upon those interested in the hospital, who knew what their work was, to take the opportunity afforded them by an occasion of that sort to show that they did greatly appreciate the work done by the Committee, work without which the hospital could not continue to be the great boon it was to the poor neighbourhood in which it was situated. (Applause.)

Mr. C. J. Thomas, in responding, pointed out the great growth which had taken place in the work done by the hospital during the last 10 years. In 1894, he said, the income was £6,500. Last year it was £16,600, but it must be remembered that that year was a specially good one, as during 1903 they had received a donation of £1,000 from Lord Portman, another of a like amount from Lord Howard de Walden, and one of £2,000 from King Edward's Hospital Fund. (Applause.) The in-patients in 1894 had numbered 781, and the out-patients 16,033; whilst in 1903 the numbers were 1,270 and 34,111 respectively, or nearly double in both cases.

The collection resulting from the dinner amounted to £2,800.

